# The genus *Bebryce* (Cnidaria, Octocorallia, Plexauridae) at Japan, with descriptions of three new species

**DOI:** 10.3897/zookeys.587.8188

**Published:** 2016-05-10

**Authors:** Asako K. Matsumoto, Leen P. van Ofwegen

**Affiliations:** 1 Planetary Exploration Research Center (PERC), Chiba Institute of Technology (Chitech), Tsudanuma 2-17-1, Narashino, Chiba 275-0016, Japan; 2 Naturalis Biodiversity Center, Darwinweg 2, P.O. Box 9517, 2300 RA Leiden, The Netherlands

**Keywords:** Anthozoa, taxonomy, new records, subtropical, temperate, deep water

## Abstract

Three new deep-water species of *Bebryce* from Japan are described and depicted using Scanning Electron Microscopy: *Bebryce otsuchiensis* **sp. n**., *Bebryce rotunda* **sp. n.**, and *Bebryce satsumaensis* **sp. n.** *Bebryce studeri* Whitelegge, 1897, was reported from Japanese waters for the first time, bringing the total of Japanese *Bebryce* species to six. Five of these six species seem to be endemic to Japanese waters and all occur in deep water up to 213 m. A key to the *Bebryce* species is presented.

## Introduction

*Bebryce* Philippi, 1841, is a genus of octocorals, which is distributed in tropical to subtropical waters in the Atlantic and Indo-Pacific Oceans. Two Japanese endemic subtropical deep-water species of *Bebryce* have been reported from the Ogasawara Islands (= Bonin Islands), both with rosettes with warty, rounded, or bristle-like projections: *Bebryce bocki* Aurivillius, 1931 and *Bebryce boninensis* Aurivillius, 1931. These two species have been re-described in a revision by Bayer and Ofwegen (2016), in which they remained the only *Bebryce* species described from Japanese waters. Meanwhile, other species have been reported from Japan, and *Bebryce bocki* has been reported outside Japan (Bayer and Ofwegen 2016).

Here we present three additional, new species, and report the finding of *Bebryce studeri* Whitelegge, 1897 in Japanese waters, a species previously known from Funafuti, New Caledonia, Indonesia, and the Philippines (Bayer and Ofwegen 2016). *Bebryce bocki* seems to be the most common *Bebryce* species in Japanese waters, whereas *Bebryce boninensis* was never found again.

## Material and methods

**Figure 1.**
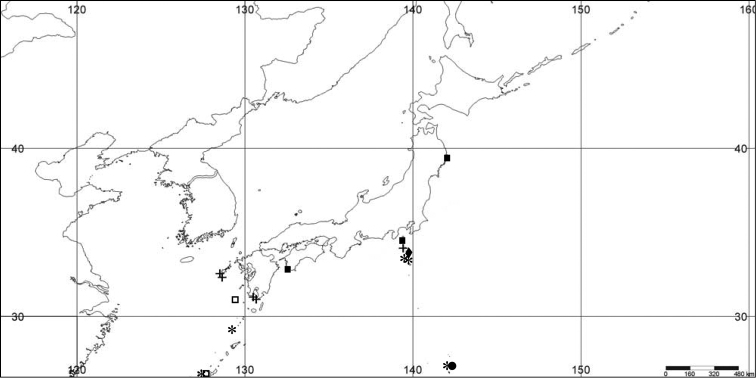
Distribution of *Bebryce bocki* (*), *Bebryce boninensis* (●), *Bebryce otsuchiensis* sp. n. (■), *Bebryce rotunda* sp. n. (◆), *Bebryce satsumaensis* sp. n., (+), and *Bebryce studeri* (□).

Material was collected by dredging, trawling or fishing net onboard research vessels *RV Tansei-maru*, University of Tokyo and Japan Agency for Marine-earth Science and Technology, *RV Yayoi*, the University of Tokyo, *RV Shinyo-maru*, Tokyo University of Marine Science and Technology, and the commercial fishing boat *Kiryo-maru* during the years 2003–2009. Depths of each station are converted to depth range in meters from shallow to deep, also when it is towed from deep to shallow if that would be indicated on the sampling label with original provenance data. We also examined historical museum material of the Zoological Museum University of Copenhagen, Denmark (ZMUC); University Museum of University of Tokyo, Japan (UMUT); and type material of *Bebryce bocki* and *Bebryce boninensis* of the Museum of Evolution, Uppsala, Sweden (UUZM) (Figure 1). Specimens were collected from a depth between 67.1 and 213 m.

Of each specimen, a small piece of the distal part of a branch was dissolved in a 4% household bleach solution to isolate sclerites. These sclerites were washed with demineralised water, dried on a hot plate, mounted on SEM stubs, and coated with Pd/Au for SEM imaging. For this, either a JEOL JSM6490LV scanning electron microscope was operated at high vacuum at 10 kV, or a JEOL JSM6510LA scanning electron microscope with a Quick Carbon Coater SC-701C, SANYU ELECTRON was used. For terminology, see Bayer et al. (1983).

Descriptions of old Japanese material collected by Japanese used “hiro” (Japanese fathom) as the depth unit. One Japanese fathom (hiro) is usually 1.43 m, occasionally 1.51 m, whereas, it is 1.818 m for the length unit on land. The old depth unit fathom is also converted to 1.8288 m. When it was not clear whether the collector used fathom or hiro, the converted depth has wider ranges.

All new type material is stored in ethanol and deposited in the Cnidaria collection (RMNH Coel.) of Naturalis Biodiversity Center, Leiden, the Netherlands (NBC).

### Abbreviations

AKM
 Asako K. Matsumoto collection, Planetary Exploration Research Center (PERC), Chiba Institute of Technology (Chitech), Japan 


BIK
 The Biological Institute on Kuroshio, Kochi, Japan 


NBC (RMNH)
Naturalis Biodiversity Center, formerly Rijksmuseum van Natuurlijke Historie Leiden, The Netherlands 


ME (UPSZTY (UUZM))
Museum of Evolution, Uppsala, Sweden 


UMUTZ
University Museum of the University of Tokyo, Japan 


ZMUC
Zoological Museum University of Copenhagen, Denmark 


### Key to the Japanese species of *Bebryce*

**Table:** 

1	Rosettes with bristle-like projections	**2**
–	Rosettes cup-shaped	**4**
2	Calycular margins without modified rosettes	***Bebryce boninensis* Aurivillius, 1931**
–	Calycular margins with modified rosettes	**3**
3	Calycular margins with asymmetrical rosettes not strongly modified	***Bebryce studeri* Whitelegge, 1897**
–	Calycular margins with spindles with blade	***Bebryce bocki* Aurivillius, 1931**
4	Coenenchymal sclerites include tuberculate disks with central process	***Bebryce rotunda* sp. n.**
–	Coenenchymal sclerites 4-6-rayed stellate plates	**5**
5	Rosettes with slightly serrated rim with spines	***Bebryce satsumaensis* sp. n.**
–	Rosettes with slightly serrated rim with blunt processes	***Bebryce otsuchiensis* sp. n.**

## Systematic part

### Bebryce bocki

Taxon classificationAnimaliaAlcyonaceaPlexauridae

Aurivillius, 1931

Figures 1 , 2a

Bebryce brocki Aurivillius, 1931: 194, fig. 38, pl. 4 fig. 4; erroneous original spelling for bocki, in honor of Sixten Bock’s expedition to the Bonin Islands, Japan.
Bebryce bocki ; Matsumoto 2014: Table 1; Bayer and Ofwegen 2016: 308.
Bebryce boninensis ; Matsumoto et al. 2007: table 1.

#### Material examined.

Holotype UPSZTY2181 (UUZM84), East of Chichijima I., Ogasawara Is. (Bonin Is.), Japan, depth 120 m (100 m in Aurivillius 1931), coll. Dr. Sixten Bock, 1 August 1914; RMNH Coel. 42080 (AKM 806) Off Takarajima I., Tokara Is., Japan, East China Sea, 29°14.6410'N, 129°07.8392'E, depth 156 m, *RV Tansei-maru*, KT07-2 cruise, st. DT5 (D8), coll. H. Yokose, 2 March 2007; AKM1407, West of Chichijima I., Ogasawara Is. (Bonin Is.), Japan, 27°01.395'N, 142°07.412'E – 27°01.360'N, 142°07.467'E, depth range 139–144 m, *RV Tansei-maru*, KT09-02, st. TW01-01, coll. A.K. Matsumoto, 19 March 2009; AKM1445, same data as AKM1407; AKM 878, off Hachijo Jima I., Izu Is., Japan, 33°20.9082'N, 139°41.1841'E – 33°21.0775'N, 139°40.4931'E, depth range 185–213 m, *RV Tansei-maru*, KT07-31, st. 14 (L-7-200), chain bag dredge, coll. A.K. Matsumoto, 26 November 2007; AKM 251(BIK-G878), off Hachijo Jima I., Izu Is., Japan, 33°26.0'N, 139°41.9'E – 33°26.1'N, 139°41.6'E, depth range 160–190 m, *RV Shinyo-maru*, KS03 cruise, st. 17, coll. A.K. Matsumoto, 21 October 2003; AKM 264 (BIK-G00902), off Hachijojima I., Izu Is., Japan, 33°26.3'N, 139°42.3'E – 33°26.5'N, 139°42.0'E, depth range 157–172 m, *RV Shinyo-maru*, KS03 cruise, st. 18, coll. A.K. Matsumoto, 21 October 2003; AKM 294 (BIK-G00907), off Hachijojima I. Izu Is., Japan, 33°26.8'N, 139°42.7'E – 33°27.0'N, 139°42.4'E, depth 170–176 m, *RV Shinyo-maru*, KS03 cruise, st. 19, coll. A.K. Matsumoto, 21 October 2003; AKM 1334, off Kerama Is. Japan, East China Sea, 26°00.55'N, 127°12.87'E – 26°00.66'N, 127°12.61'E, depth range 97–100 m, *RV Tansei-maru*, KT08-33, KR-3, chain bag dredge, coll. A.K. Matsumoto, 18 December 2008.

#### Diagnosis.

*Bebryce* with rosettes with warty, rounded, or bristle-like projections. Those of calycular margin asymmetrically developed, with strong projecting blade. Coenenchymal sclerites are thick, warty disks.

#### Remarks.

Apparently this is the most common *Bebryce* species in sub-tropical to temperate Japanese waters, in a depth range of 97–213 m.

**Figure 2.**
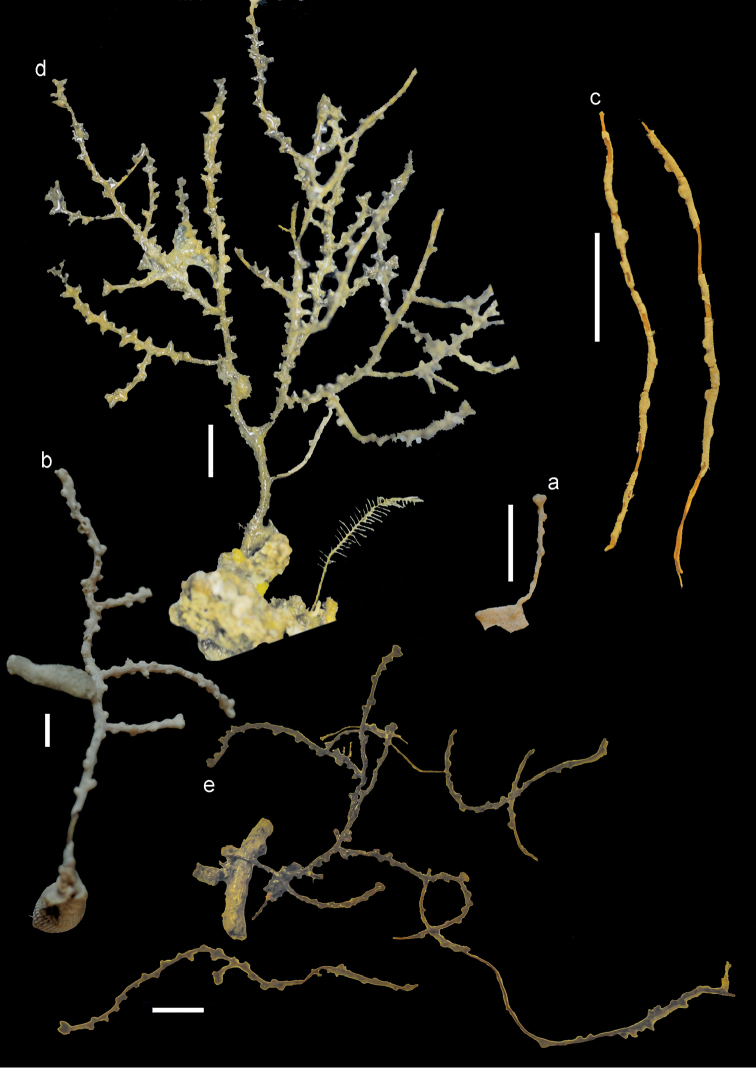
**a** *Bebryce bocki* Aurivillius, 1931, holotype (UPSZTY2181 (UUZM84)) **b** *Bebryce boninensis* Aurivillius, 1931, holotype (UPSZTY2166 (UUZM69)) **c** *Bebryce otsuchiensis* sp. n., holotype (RMNH Coel. 42072) **d** *Bebryce rotunda* sp. n., holotype (RMNH Coel. 42076) **e** *Bebryce satsumaensis* sp. n., holotype (RMNH Coel. 42077). Scales: 1 cm.

### Bebryce boninensis

Taxon classificationAnimaliaAlcyonaceaPlexauridae

Aurivillius, 1931

Figures 1 , 2b

Bebryce boninensis Aurivillius, 1931: 200, fig. 39, pl. 4 fig. 3 (Bonin Is., Japan); Matsumoto 2014: Table 1; Bayer and Ofwegen 2016: 308.
Bebryce boninensis NOT Bebryce boninensis; Matsumoto et al. 2007: table 1 = Bebryce bocki.

#### Material examined.

Holotype UPSZTY2166 (UUZM69), ENE from Anojima I. (Anijima I. or Anejima I.), Ogasawara Is. (Bonin Is.), Japan, depth 150 m (100 fathoms in Aurivillius 1931), coll. Dr. Sixten Bock, 15 August 1914.

#### Diagnosis.

*Bebryce* with rosettes with warty, rounded, or bristle-like projections. Calycular margins without specialized sclerites. Coenenchymal sclerites are thick, warty disks.

#### Remarks.

It cannot be excluded that this species is synonymous with *Bebryce bocki*. Its sclerites are very similar and it only differs in lacking the asymmetrical rosettes at the calyx margin. These sclerites may perhaps fall off easily, which would explain why the species was never reported again. The distance between Chichijima Island (type locality of *Bebryce bocki*) and Anijima Island (type locality of *Bebryce boninensis*) is ca. 800 m within the Anijima Strait. The recorded depth of *Bebryce boninensis* (150 m) is within the depth range of *Bebryce bocki* (97–213 m). As collecting efforts at the Bonin Islands have been limited, the two species are still considered separate in the present study. Re-examination of the material studied by Matsumoto et al. (2007), proved to be *Bebryce bocki*.

### Bebryce otsuchiensis sp. n.

Taxon classificationAnimaliaAlcyonaceaPlexauridae

http://zoobank.org/E4DF6D06-CC14-4469-A19D-3428D656E789

**Figure 3.**
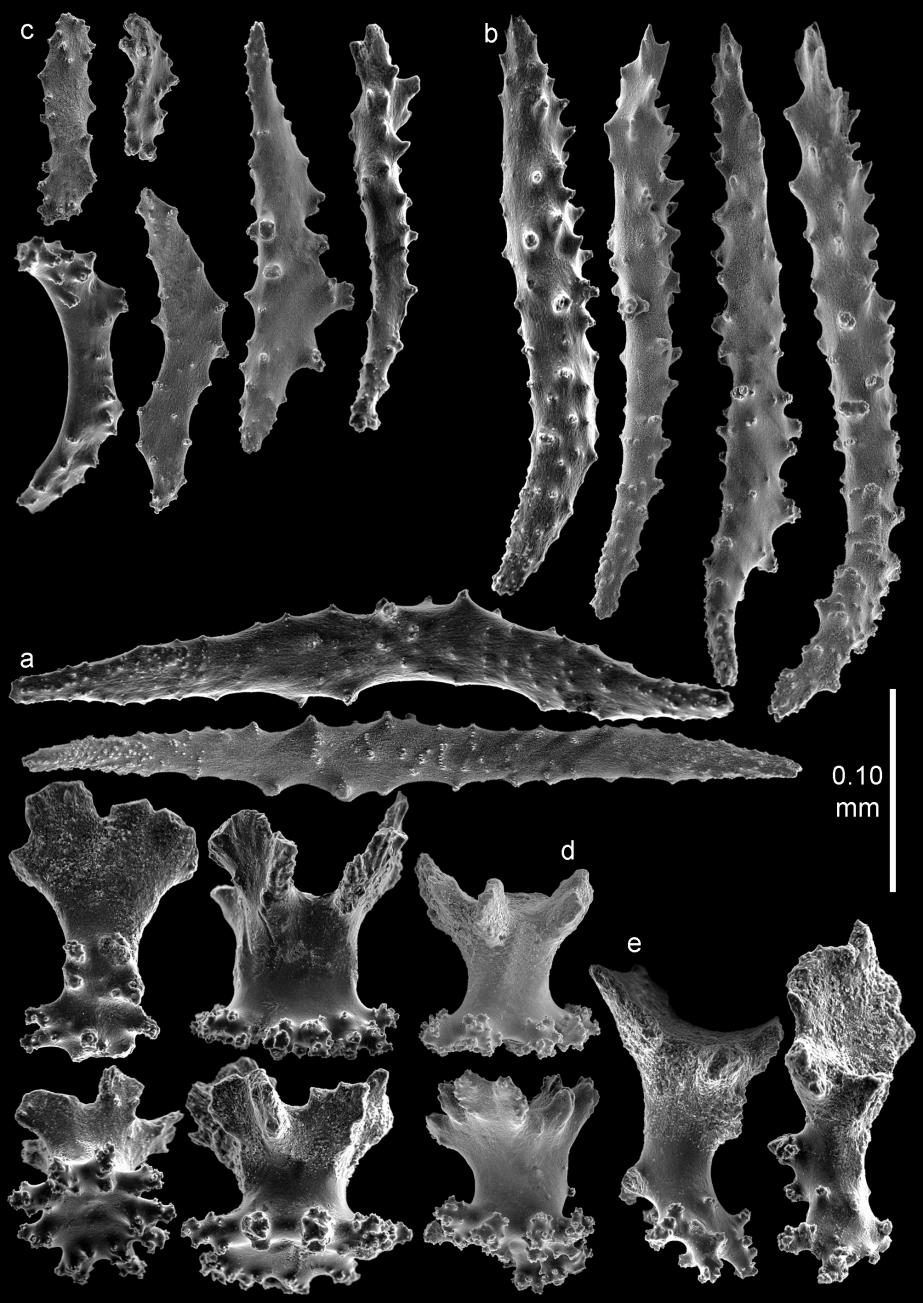
*Bebryce otsuchiensis* sp. n., holotype (RMNH Coel. 42072) **a** collaret spindles **b** point spindles **c** tentacle sclerites **d** rosettes of outer surface of coenenchyme **e** asymmetrical rosettes from calyx rim.

**Figure 4.**
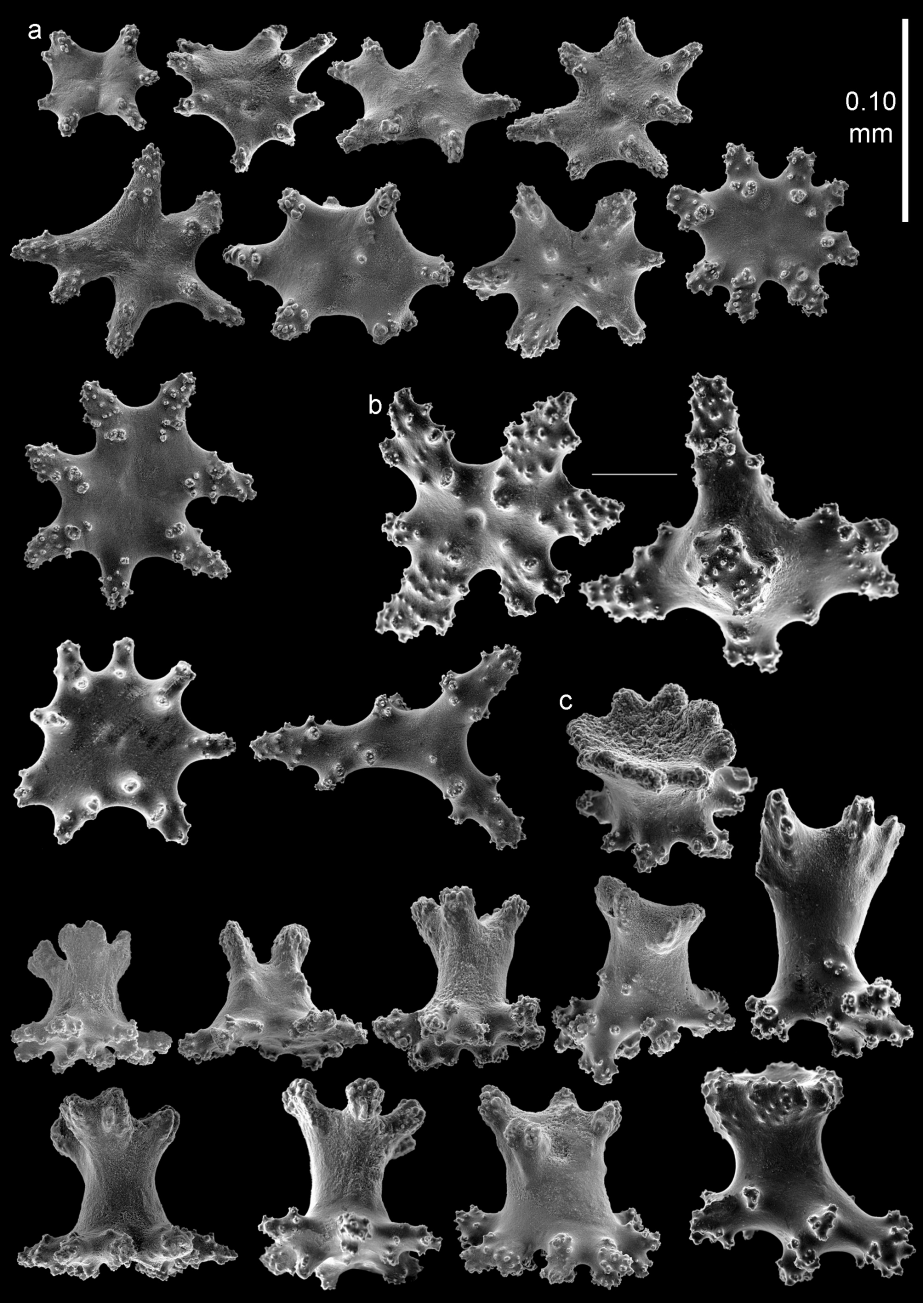
*Bebryce otsuchiensis* sp. n., holotype (RMNH Coel. 42072) **a–b** 3–6 rayed stellate plates of deeper layer of coenenchyme **c** rosettes of outer surface of coenenchyme.

**Figure 5.**
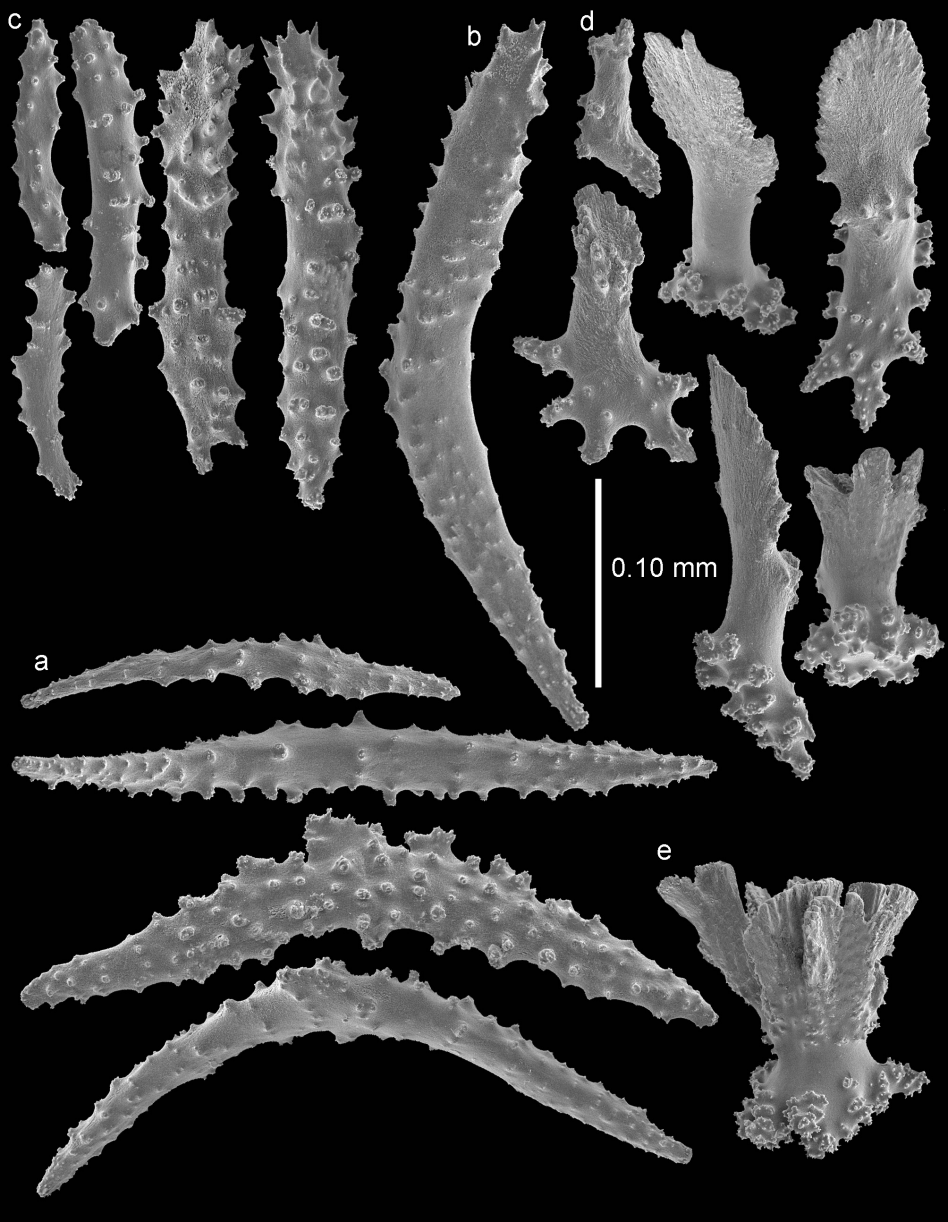
*Bebryce otsuchiensis* sp. n., paratype (RMNH Coel. 42074) **a** collaret spindles **b** point spindle **c** tentacle sclerites **d** asymmetrical rosettes from calyx rim **e** rosette of outer surface of coenenchyme.

**Figure 6.**
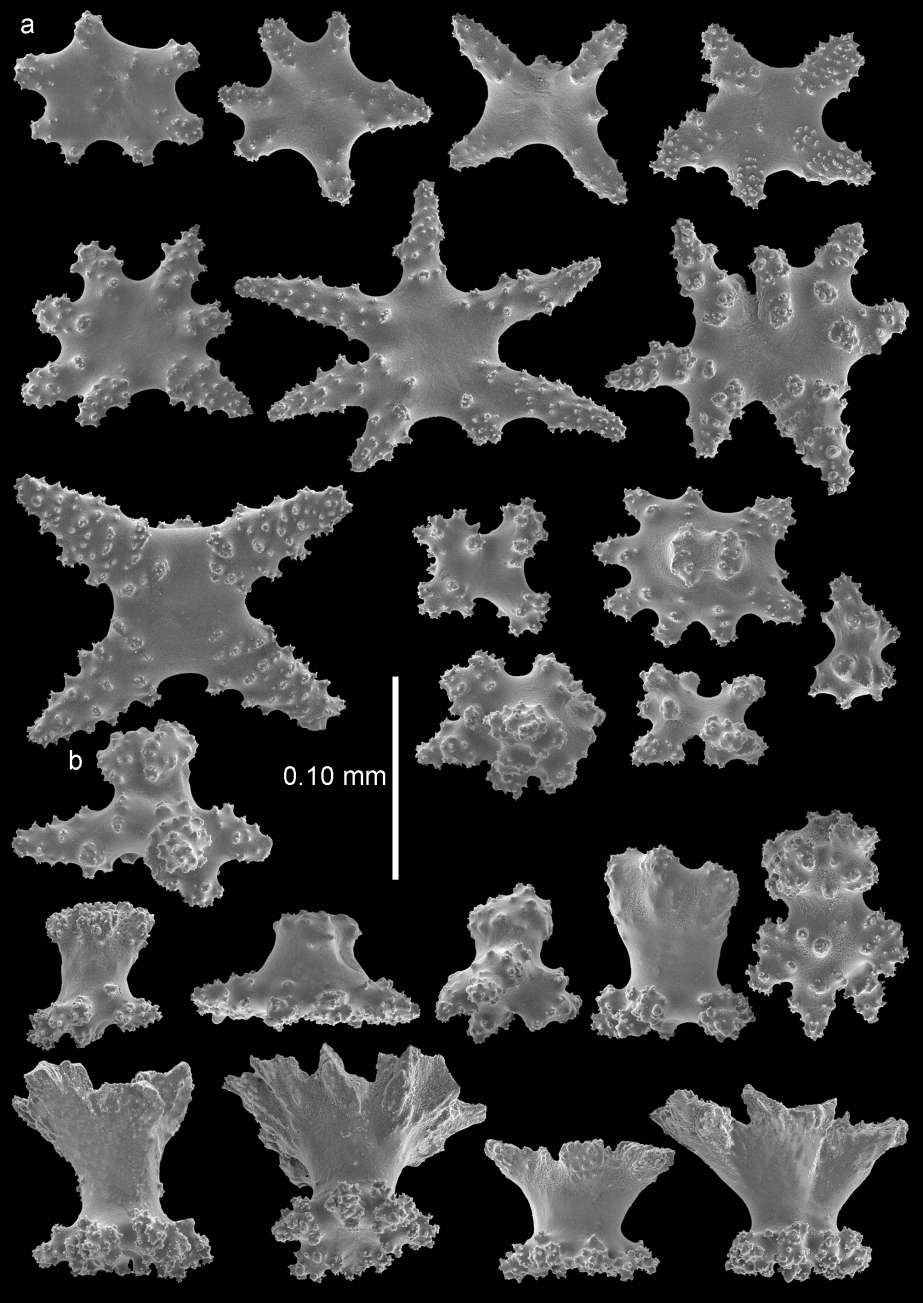
*Bebryce otsuchiensis* sp. n., paratype (RMNH Coel. 42074) **a** 3–6 rayed stellate plates of deeper layer of coenenchyme **b** rosettes of outer surface of coenenchyme.

Figures 1 , 2c , 3 , 4 , 5 , 6

#### Material examined.

Holotype RMNH Coel. 42072 (AKM 703), Entrance of Otsuchi Bay, Iwate Prefecture, Japan, 39°21.8052'N, 142°00.0750'E – 39°22.0672'N, 141°59.9619'E, depth 67–81 m, *RV Yayoi*, st. 2, 1 m biological dredge, coll. A.K. Matsumoto, 23 May 2006; paratypes RMNH Coel. 42073 (AKM 531), Otsuki, Tosa, Kochi Prefecture, Japan, 32°34.14'N, 132°48.59'E – 32°34.18'N, 132°47.53'E, depth range 117–125 m, local fishermen’s boat *Kiryo-maru*, st. 2, coral net, coll. A.K. Matsumoto, 7 October 2004; RMNH Coel. 42074 (AKM 1628), Otsuki, Tosa, Kochi prefecture, Japan, 32°37.66'N, 132°50.44'E – 32°37.56'N, 132°47.88'E, depth 114 m, local fishermen’s boat *Kiryo-maru*, st. 1, coral net, coll. A.K. Matsumoto, 7 October 2004; RMNH Coel. 42075 (AKM 943), Toshima I., Izu Is., Japan, 34°33.1102'N, 139°17.4102'E – 34°33.6524'N, 139°17.6725'E, depth 143 m, *RV Tansei-maru*, KT07-31 cruise (Kuramochi leg), st. 22 (L-3-100), chain bag dredge, coll. A.K. Matsumoto, 27 November 2007.

#### Description.

The holotype RMNH Coel. 42072 consists of two branches, both 4 cm long. (Figure 2c). The calyces are placed spirally all around the slender branches, which are about 1 mm wide. The dome-shaped calyces are about 1 mm wide and high.

The anthocodiae are armed with a crown and points consisting of a transverse crown with curved, rather smooth spindles up to 0.40 mm long (Figure 3a) and eight points formed by spindles 0.35 mm long (Figure 3b) placed in a chevron-like pattern beneath the tentacles. These spindles have simple tubercles and a distal spiny end. The tentacles contain flattened, dragon wing sclerites up to 0.2 mm long (Figure 3c).

The sclerites of the outer surface of coenenchyme and calyces are rosettes consisting of a cup-shaped thorny projection arising from a warty base. Several of these are up to 0.10 mm long and have a widely flared calyx part of about 0.10 mm in greatest diameter with slightly serrated rim with a few blunt processes, joined by a smooth, slender stem to a warty base narrower than the calyx (Figure 3d); others do not flare out (Figure 4c). The rosettes become asymmetrical toward the calycular apertures (Figure 3e), with the calyx margin becoming elongated and forming a blade-like process that projects from the surface and surrounds the calycular aperture. These sclerites are up to 0.20 mm long.

The deeper layer of coenenchyme contains stellate plates, 3–6 rayed forms up to 0.15 mm in the greater diameter, with a central process (Figure 4a). Most are weakly tuberculated (Figure 4a) but several are more tuberculated towards the end of the rays (Figure 4b).

**Colour.** The holotype is light brown.

#### Etymology.

Named after the type locality, Otsuchi Bay.

#### Variation.

RMNH Coel. 42074 (AKM 1628) has slightly wider point sclerites, collaret spindles heavier tuberculate, and more tuberculate stellate plates (Figures 5–6).

#### Comparisons.

The species mostly resembles *Bebryce harpy* Grasshoff, 1999, regarding the blunt processes of the rosettes. It differs in overall having less tuberculate sclerites.

#### Remarks.

This is the northernmost species of *Bebryce*. It has a very wide distribution from North to South Japan, and is only found in the warm Kuroshio Current area, in the depth range 67–143 m. This species also represents the northernmost record of the genus *Bebryce*, and the first from north of 39°N latitude.

### Bebryce rotunda sp. n.

Taxon classificationAnimaliaAlcyonaceaPlexauridae

http://zoobank.org/57ED4C53-5C51-4089-B26C-230EB84A1C79

**Figure 7.**
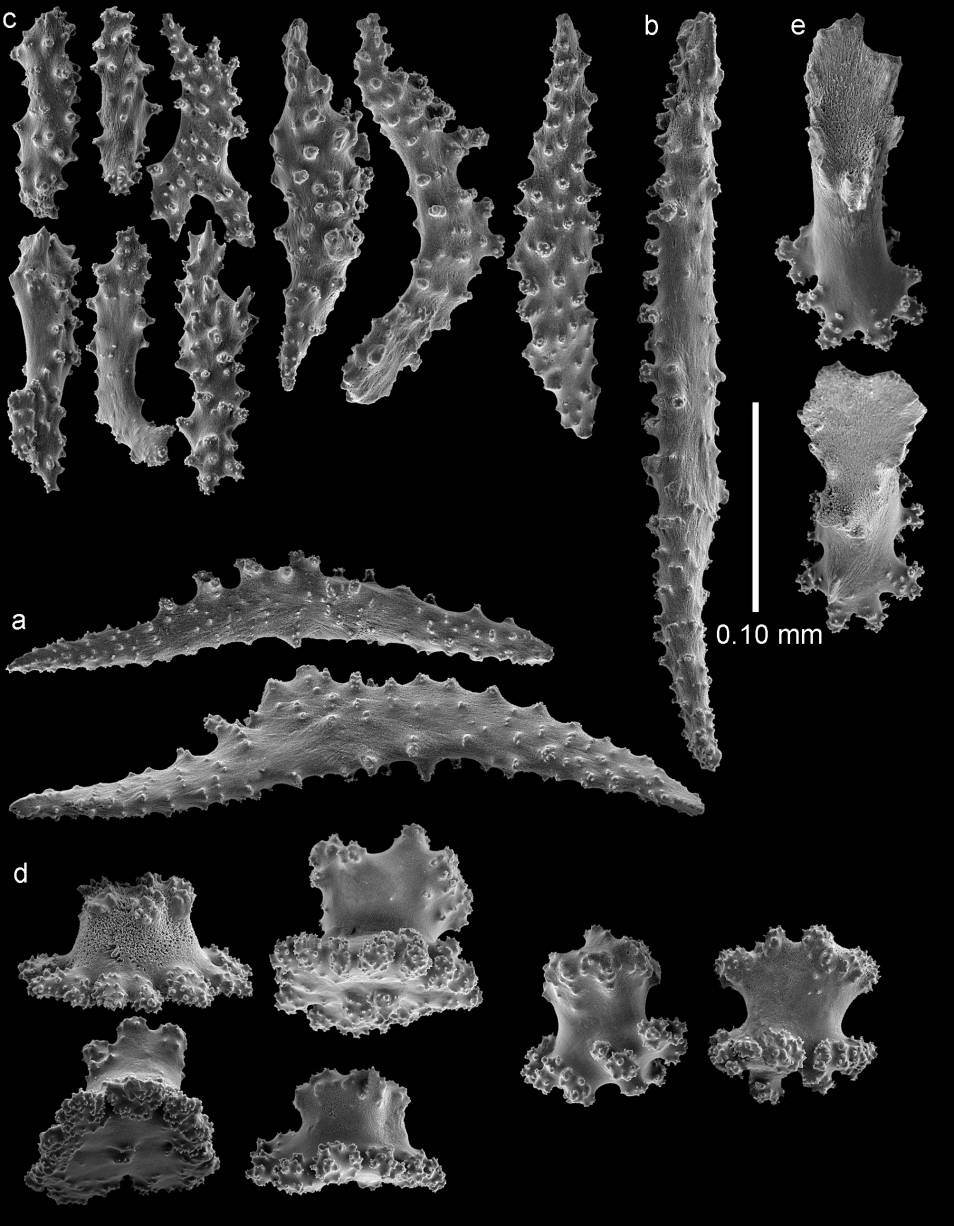
*Bebryce rotunda* sp. n., holotype (RMNH Coel. 42076) **a** collaret spindles **b** point spindle **c** tentacle sclerites **d** rosettes of outer surface of coenenchyme **e** asymmetrical rosettes from calyx rim.

**Figure 8.**
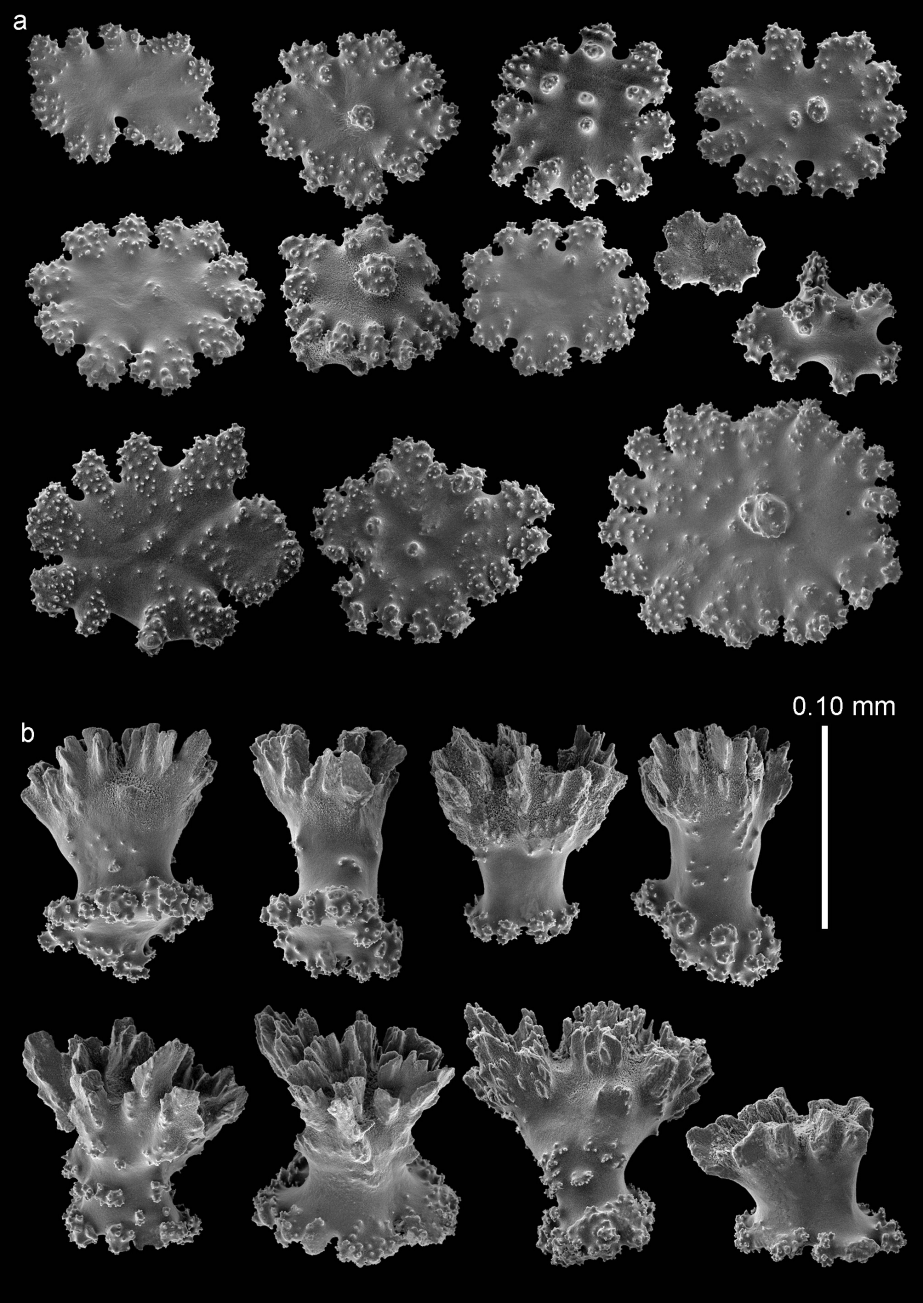
*Bebryce rotunda* sp. n., holotype (RMNH Coel. 42076) **a** tuberculate disks of deeper layer of coenenchyme **b** rosettes of outer surface of coenenchyme.

Figures 1 , 2d , 7 , 8

#### Material examined.

Holotype RMNH Coel. 42076 (AKM 881), Hachijo I., Izu Is., Japan, 33°20.9082'N, 139°41.1841'E – 33°21.0775'N, 139°40.4931'E, depth range 185–213 m, *RV Tansei-maru*, KT07-31 cruise (Kuramochi leg), st. 14 (L-7-200), chain bag dredge, coll. A.K. Matsumoto, 26 November 2007.

#### Description.

The holotype RMNH Coel. 42076 (AKM 881) is a sparsely branched colony 10 cm long (Figure 2d). The calyces are placed spirally all around the slender branches, which are about 1 mm wide. The stem is 2 cm wide. The dome-shaped calyces are about 2 mm wide and high.

The anthocodiae are armed with a crown and points consisting of a transverse crown with curved spindles up to 0.35 mm long (Figure 7a) and eight points formed by spindles 0.35 mm long (Figure 7b) placed in a chevron-like pattern beneath the tentacles. These spindles have simple tubercles and a distal spiny end. The tentacles contain flattened, dragon wing sclerites up to 0.2 mm long (Figure 7c).

The sclerites of the outer surface of coenenchyme and calyces are rosettes consisting of a cup-shaped thorny projection arising from a warty base. These rosettes are 0.10 mm tall, have a flared calyx part about 0.10 mm in greatest diameter with blunt processes (Figure 7d) or the rim of the cups is formed several strong, laciniated projections (Figure 8b). Toward the calycular apertures the rosettes become asymmetrical (Figure 7e), with the margin of the calyx becoming much elongated forming a blade-like process that projects from the surface and surrounds the calycular aperture. These sclerites are up to 0.15 mm long.

The plates of the inner coenenchyme are tuberculate disks up to about 0.15 mm in diameter with tuberculate rim and central process on one surface (Figure 8a).

**Colour.** The holotype is creme.

#### Etymology.

From the Latin *rotundus*, wheel-shaped, round, referring to the round disks of the coenenchyme.

#### Comparisons.

Bayer and Ofwegen (2016) mentioned only two species with tuberculate disks and cup-shaped rosettes, *Bebryce* species A and *Bebryce thomsoni* Nutting, 1910. The present species differs from these two by having disks with a smooth central part and a small process in the middle, while those of *Bebryce* species A and *Bebryce thomsoni* have tubercles all over the disk.

### Bebryce satsumaensis sp. n.

Taxon classificationAnimaliaAlcyonaceaPlexauridae

http://zoobank.org/C2586676-9927-4B9D-8C9E-B1E509C1EB29

**Figure 9.**
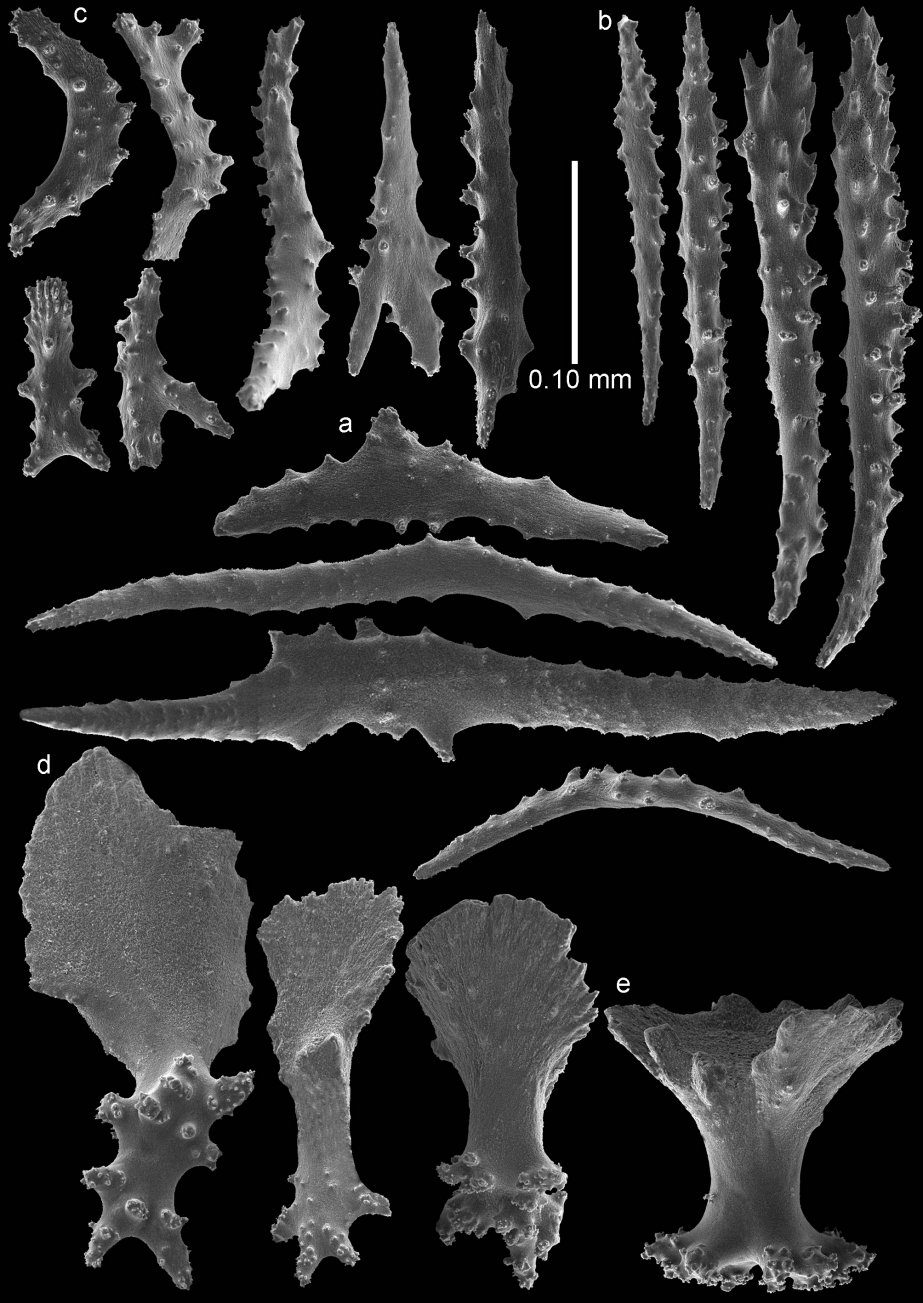
*Bebryce satsumaensis* sp. n., holotype (RMNH Coel. 42077) **a** collaret spindles **b** point spindles **c** tentacle sclerites **d** asymmetrical rosettes from calyx rim **e** rosette of outer surface of coenenchyme.

**Figure 10.**
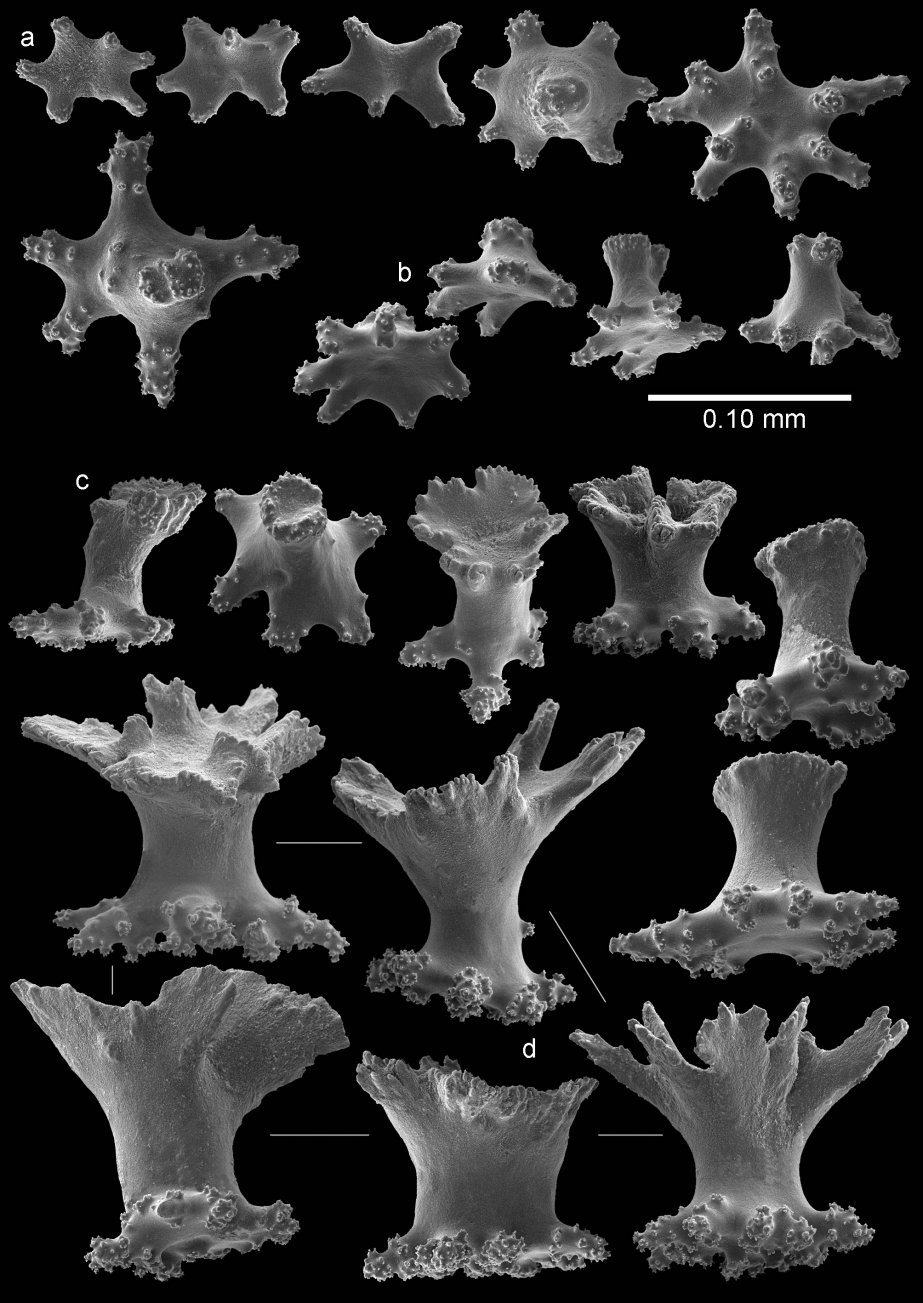
*Bebryce satsumaensis* sp. n., holotype (RMNH Coel. 42077) **a** 3–6 rayed stellate plates of deeper layer of coenenchyme **b**–**d** rosettes of outer surface of coenenchyme.

Figures 1 , 2e , 9 , 10

#### Material examined.

Holotype RMNH Coel. 42077 (AKM760), off Sata-misaki Cape, Kagoshima Prefecture, Japan, 30°56.0025'N, 130°44.2299'E – 30°56.2953'N, 130°43.3981'E, depth range 116–120 m, *RV Tansei-maru*, KT07-1 cruise, st. SM-1, chain bag dredge, coll. A.K. Matsumoto, 23 February 2007; paratypes RMNH Coel. 42078 (AKM 1629), off Sata-misaki Cape, Kagoshima Prefecture, Japan, 31°00.50'N, 130°35.09'E – 31°01.3211'N, 130°34.6509'E, depth range 178–189 m, *RV Tansei-maru*, KT07-1 cruise, st. SM-2, coll. A.K. Matsumoto, 23 February 2007; UMUTZ-CnidG-49, off Kozushima I., Izu Is., Sagami Bay, Japan, Ohnoura-maru, 24 August 1893; UMUTZ-CnidG-90, Yamagawa, below Kaimon-dake mt., Kagoshima Bay (Kagoshima Prefecture), Japan, depth 70 Japanese fathoms (100–106 m), Prof. Mitsukuri & Hara Satsuma Exp., long line, coll. S. Azuma, 8 April 1896; UMUTZ-CnidG-91, Odawara, Kanagawa Prefecture, Japan, depth 120 hiro (Japanese fathoms (171–181 m)), coll. I. Ijima, August 1895; ZMUC-ANT-000645 (ZMUC 120604-39), East China Sea, 32°15'N, 128°12'E, depth 90 fms (165 m), hard bottom, gear: Shveber, *Hyateri-maru*, Dr. Th. Mortensen’s Pacific Expedition 1914–1915, coll. Dr. Th. Mortensen, 15 May 1914; RMNH Coel. 42079 (AKM 1092), Shin-sone bank, Danjo Is., Japan, East China Sea, 31°54.61'N, 128°19.56'N, – 31°54.64'N, 128°19.41'E, depth range 200–210 m , *RV Tanisei-maru*, KT08-3(Oji leg), st. GT02(2), ORI-TI chain bag dredge, coll. A.K. Matsumoto, 7 March 2008.

#### Description.

The holotype RMNH Coel. 42077 consists of a sparsely branched colony about 5 cm long and a few loose branches (Figure 2e). The calyces are placed spirally all around the slender branches, which are about 1 mm wide. The dome-shaped calyces are about 1 mm wide and high.

The anthocodiae are armed with a crown and points consisting of a transverse crown with curved, rather smooth spindles up to 0.45 mm long (Figure 9a) and eight points formed by spindles 0.3 mm long (Figure 9b) placed in a chevron-like pattern beneath the tentacles. These spindles have simple tubercles and a distal spiny end. The tentacles contain flattened, dragon wing sclerites up to 0.2 mm long (Figure 9c).

The sclerites of the outer surface of coenenchyme and calyces are rosettes consisting of a cup-shaped thorny projection arising from a warty base. Several, about 0.15 mm tall, have a widely flared calyx part about 0.15 mm in greatest diameter with slightly serrated rim with some spines, joined by a smooth, slender stem to a warty base narrower than the calyx (Figures 9e, 10d). Others have a less serrated rim which does not flare out (Figure 10c). Toward the calycular apertures the rosettes become asymmetrical (Figure 9d), with the margin of the calyx becoming much elongated, forming a blade-like process that projects from the surface and surrounds the calycular aperture. These sclerites are up to 0.25 mm long.

The deeper layer of coenenchyme contains stellate plates, 4–6 rayed forms up to 0.10 mm in the greater diameter, with a central process (Figure 10a). Stellate sclerites with a prominent, thorny central process, intermediate in form between the cup-shaped outer forms and the stellate plates of the deeper coenenchyme are not uncommon (Figure 10b).

**Colour**. The holotype is brown.

#### Etymology.

Named after the type locality, Satsuma (old name of Kagoshima prefecture).

#### Comparisons.

The rosettes with weakly serrate rim and few spines are unique for this species within the genus. *Bebryce brunnea* (Nutting, 1908) and *Bebryce cofferi* Bayer and Ofwegen, 2016 resemble this species but have rosettes with more serrate rim.

#### Remarks.

ZMUC-ANT-000645 (ZMUC 120604-39) was listed as ?*Bebryce* sp. in Matsumoto 2014.

The species occurs in South Japan up to Sagami Bay, in the depth range 100–210 m.

### Bebryce studeri

Taxon classificationAnimaliaAlcyonaceaPlexauridae

Whitelegge, 1897

Figures 1 , 11

#### Material examined.

AKM1280, off Kerama Is., Okinawa Prefecture, Japan, East China Sea, 26°04.59'N, 127°27.70'E – 26°04.56'N, 127°27.95'E, depth range 153–160 m, *RV Tansei-maru*, KT08-33 cruise (Oji leg), KR-7, chain bag dredge, coll. A.K. Matsumoto, 16 December 2008; UMUTZ-Cnid G103, coral ground, Uji Is., Satsuma, Kanogshima Prefecture, Japan, depth ca. 80 fms (114–121 m), coll. K. Kinoshita, June 1908.

#### Diagnosis.

*Bebryce* with rosettes with warty, rounded, or bristle-like projections. Those of calycular margin asymmetrically developed, with weakly developed projecting blade. Coenenchymal sclerites are warty disks.

#### Remarks.

This is the first record of this species for Japan, where it is limited to the South of Japan, East China Sea. The depth record of previous studies is 23–113 m, from Funafuti, New Caledonia, Indonesia, Philippines, and Papua New Guinea (Bayer and Ofwegen 2016). This study recorded a deeper depth range, namely 114–160 m.

**Figure 11.**
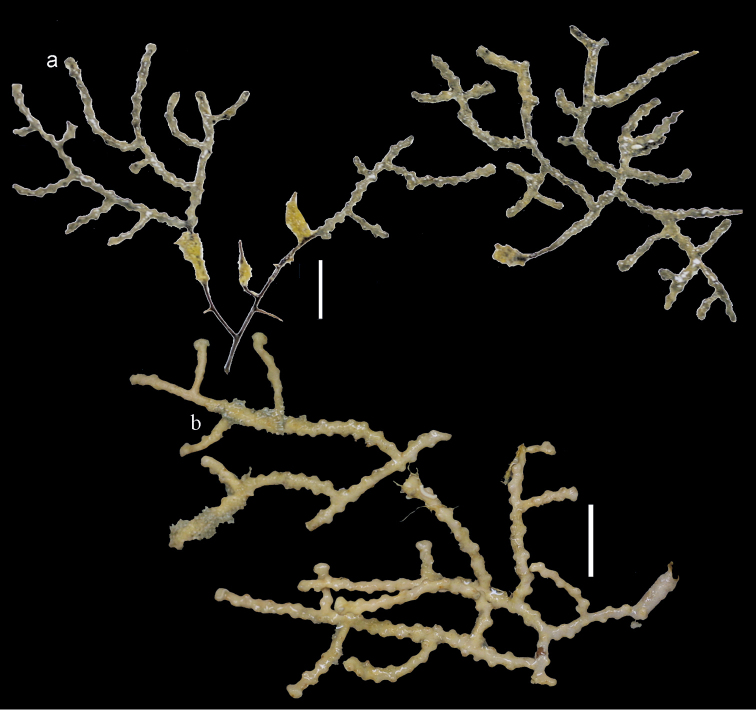
*Bebryce studeri* Whitelegge, 1897 **a** AKM1280 **b** UMUTZ-Cnid G103. Scales: 1 cm.

## Supplementary Material

XML Treatment for Bebryce bocki

XML Treatment for Bebryce boninensis

XML Treatment for Bebryce otsuchiensis

XML Treatment for Bebryce rotunda

XML Treatment for Bebryce satsumaensis

XML Treatment for Bebryce studeri
